# Spontaneous hypothermia on intensive care unit admission is a predictor of unfavorable neurological outcome in patients after resuscitation: an observational cohort study

**DOI:** 10.1186/cc9077

**Published:** 2010-06-23

**Authors:** Alexander W den Hartog, Anne-Cornélie JM de Pont, Laure BM Robillard, Jan M Binnekade, Marcus J Schultz, Janneke Horn

**Affiliations:** 1 Department of Intensive Care Medicine, Academic Medical Center, University of Amsterdam, Meibergdreef 9, Amsterdam, 1105 AZ, The Netherlands; 2 Department of Intensive Care Medicine, Academic Medical Center, University of Amsterdam, Laboratory of Experimental Intensive Care and Anesthesiology (LEICA), HERMES Critical Care Group, Meibergdreef 9, Amsterdam, 1105 AZ, The Netherlands

## Abstract

**Introduction:**

A large number of patients resuscitated for primary cardiac arrest arrive in the intensive care unit (ICU) with a body temperature < 35.0°C. The aim of this observational cohort study was to determine the association between ICU admission temperature and neurological outcome in this patient group.

**Methods:**

Demographics and parameters influencing neurological outcome were retrieved from the charts of all patients resuscitated for primary cardiac arrest and treated with induced mild hypothermia in our ICU from January 2006 until January 2008. Patients were divided into two groups according to their body temperature on ICU admission: a hypothermia group (< 35.0°C) and a non-hypothermia group (≥35.0°C). Neurological outcome after six months was assessed by means of the Glasgow Outcome Score (GOS), with GOS 1 to 3 defined as unfavorable and GOS 4 to 5 as favorable. A logistic regression model was used to analyze the influence of the different parameters on neurological outcome.

**Results:**

The data of 105 consecutive patients resuscitated for primary cardiac arrest and treated with induced mild hypothermia were analyzed. Median ICU admission temperature was 35.1°C (interquartile range (IQR) 34.3 to 35.7). After six months, 61% of the patients had an unfavorable outcome (59% died and 2% were severely disabled), whereas 39% had a favorable outcome (moderate disability or good recovery). Among patients with spontaneous hypothermia on ICU admission, the percentage with unfavorable outcome was higher (69% versus 50%, *P *= 0.05). Logistic regression showed that age, acute physiology and chronic health evaluation (APACHE) II and sequential organ failure assessment (SOFA) scores and spontaneous hypothermia on ICU admission all had an increased odds ratio (OR) for an unfavorable outcome after six months. Spontaneous hypothermia had the strongest association with unfavorable outcome (OR 2.6, 95% CI (confidence interval) 1.1 to 5.9), which became even stronger after adjustment for age, presenting heart rhythm, APACHE II and SOFA scores (OR 3.8, CI 1.3 to 11.0).

**Conclusions:**

In this observational cohort study, spontaneous hypothermia on ICU admission was the strongest predictor of an unfavorable neurological outcome in patients resuscitated for primary cardiac arrest.

## Introduction

A large number of patients resuscitated for cardiac arrest arrive in the intensive care unit (ICU) with hypothermia. Since two prospective randomized multicenter trials demonstrated a beneficial effect of induced mild hypothermia on neurological outcome after cardiac arrest [1,2], one could argue that patients with spontaneous hypothermia on ICU admission might have a better outcome, since the time needed to reach the target body temperature during induced mild hypothermia is reduced. On the other hand, spontaneous hypothermia could be a sign of impaired thermoregulation and thus be related to an unfavorable outcome. The aim of the current study was to analyze whether spontaneous hypothermia on ICU admission was a predictor of neurological outcome in patients resuscitated for primary cardiac arrest.

## Materials and methods

### Study design

We performed an observational cohort study based on stored data from patients treated in our ICU in 2006 and 2007. The institutional review board approved the study and since no intervention was performed, the need for informed consent was waived.

### Study population and data sources

Data from all consecutive patients resuscitated for cardiac arrest and treated with induced mild hypothermia in our 28-bed mixed medical-surgical ICU between 1 January 2006 and 1 January 2008 were retrieved from our Patient Data Management System (PDMS, Metavision, iMDsoft, Sassenheim, The Netherlands) and from the patient's charts. Patients comatose for reasons other than cardiac arrest (drug overdose, head trauma, cerebrovascular accident or primary neurological disease) and patients with cardiac arrest due to primary hypoxemia (asphyxia, drowning or pulmonary embolism) were excluded from the analysis. Collected data included demographics, presenting heart rhythm, ICU admission temperature, Acute Physiology and Chronic Health Evaluation (APACHE) II score and Sequential Organ Failure Assessment (SOFA) score on ICU admission. Patients were divided into two groups according to their ICU admission temperature, a spontaneous hypothermia group (< 35.0°C) and a non-hypothermia group (≥35.0°C).

### Outcome

Neurological outcome was assessed with the Glasgow Outcome Score (GOS) six months after ICU admission. For this purpose, surviving patients or their relatives were interviewed by telephone. A GOS of 1 (death), 2 (vegetative state) or 3 (severe disability) was defined as unfavorable, whereas a GOS of 4 (moderate disability) or 5 (good recovery) was defined as favorable.

### Statistical analysis

Data were analyzed using the Statistical Package for Social Sciences (SPSS) for Windows, version 15.1 (SPSS, Chicago, IL, USA). Patient characteristics were analyzed using non-parametric tests. A logistic regression model was used to analyze the influence of different parameters on neurological outcome (GOS) six months after ICU admission. Variables were included in the logistic regression model if significantly associated with unfavorable neurological outcome on a univariate basis at a *P*-value of < 0.10. Co-linearity between variables was excluded prior to modeling by computing correlation with an r > 0.5 considered to indicate colinearity. The influence of ICU admission temperature on outcome was adjusted for age, presenting heart rhythm, APACHE II and SOFA scores on ICU admission. Data are presented as median and interquartile range (IQR) and as odds ratios (ORs) with 95% confidence intervals (CIs). A *P*-level of less than 0.05 was considered statistically significant.

## Results

### Study population

During the study period, 123 patients were treated with induced mild hypothermia in our ICU after resuscitation for cardiac arrest. Eighteen patients were excluded from the analysis: in 17 patients cardiac arrest was due to primary hypoxemia (asphyxia n = 8, near-drowning n = 6, pulmonary embolism n = 3) and in one patient to autointoxication with nortryptiline. The characteristics of the remaining 105 patients are summarized in Table 1. Median ICU admission temperature was 35.1°C (IQR 34.3 to 35.7). Forty-nine patients (47%) were admitted to the ICU with spontaneous hypothermia (ICU admission temperature < 35°C). This occurred significantly more frequently after resuscitation for out of hospital cardiac arrest than for in hospital cardiac arrest (53% versus 7%, *P *< 0.01). Age, gender, presenting rhythm and SOFA score on admission did not differ significantly between patients admitted to the ICU with hypothermia or non-hypothermia. Remarkably, patients with spontaneous hypothermia on ICU admission had a significantly lower APACHE score (21 versus 28, *P *= 0.03) and therefore a lower predicted mortality (38% versus 72%, *P *< 0.01).

**Table 1:** Patient characteristics

Characteristic	ICU admission temperature < 35.0°C N = 49	ICU admission temperature ≥35.0°C N = 56	*P*-value
Age, median (IQR)	64 (51 to 71)	60 (53 to 72)	0.82
Male gender, n (%)	36 (73)	42 (75)	0.86
Location of cardiac arrest			
Out of hospital, n (%)	48 (98)	42 (75)	< 0.005
Presenting rhythm			
VT/VF, n (%)	33 (67)	44 (79)	0.20
APACHE II score, median (IQR)	21 (15 to 29)	28 (21 to 32)	0.03
Predicted mortality (%)	49 (25 to 75)	70 (40 to 80)	< 0.01
SOFA score on admission, median (IQR)	8 (6 to 11)	7 (6 to 9)	0.23
Unfavorable outcome (GOS 1 to 3), n (%)	34 (69)	28 (50)	0.04

ICU, Intensive Care Unit; IQR, interquartile range; VT, ventricular tachycardia; VF, ventricular fibrillation; APACHE, Acute Physiology And Chronic Health Evaluation; SOFA, Sequential Organ Failure Assessment; GOS, Glasgow Outcome Scale.

### Outcome

Three patients were lost to follow-up. Among the remaining 102 patients, 62 (61%) had an unfavorable neurological outcome after six months: 60 patients died (GOS 1) and 2 were severely disabled (GOS 3). There were no patients remaining in permanent vegetative state (GOS 2) after six months; all these patients had died. The remaining 40 patients (39%) had a good recovery (GOS 5). An unfavorable neurological outcome occurred more frequently among patients with spontaneous hypothermia on ICU admission (69% versus 50%, *P *= 0.05). Moreover, all patients admitted to the ICU with a body temperature < 32.8°C died (n = 4). Since 98% of the hypothermic patients were resuscitated outside the hospital, this might have been a confounding factor. Therefore we separately analyzed outcome according to the location of the cardiac arrest. The results of this analysis are depicted in Table 2. Similar to the outcome in the complete group, patients resuscitated outside the hospital more frequently had an unfavorable neurological outcome if they were admitted to the ICU with spontaneous hypothermia (70% versus 45%, *P *= 0.02).

**Table 2:** Neurological outcome as a function of location of cardiac arrest

Characteristic	ICU admission temperature < 35.0°C N = 49	ICU admission temperature ≥35.0°C N = 56	*P*-value
Out of hospital, n	48	42	
Unfavorable outcome (GOS 1 to 3), n (%)	33 (69)	18 (43)	0.02
Favorable outcome (GOS 4 to 5), n (%)	14 (29)	22 (52)	
Lost to follow up, n (%)	1 (2)	2 (5)	
In hospital, n	1	14	
Unfavorable outcome (GOS 1 to 3), n (%)	1 (100)	10 (71)	NS
Favorable outcome (GOS 4 to 5), n (%)	0 (0)	4 (29)	
Lost to follow up, n (%)	0 (0)	0 (0)	

ICU, intensive care unit; GOS, Glasgow Outcome Scale.

The results of the logistic regression analysis are summarized in Table 3. Spontaneous hypothermia, age, APACHE II and SOFA scores all had an increased OR for an unfavorable neurological outcome after six months, with spontaneous hypothermia being the strongest predictor of unfavorable outcome (OR 2.6, 95% CI 1.1 to 5.9). When spontaneous hypothermia was adjusted for age, APACHE II and SOFA scores, the influence on neurological outcome was even stronger (OR 3.8, 95% CI 1.3 to 11.0). Moreover, in the subgroup of patients resuscitated outside the hospital, spontaneous hypothermia was a stronger independent predictor of unfavorable neurological outcome than in the full cohort (OR 2.9, 95% CI 1.2 to 7.0).

**Table 3:** Multivariable logistic regression analysis with unfavorable outcome (GOS 1 to 3) after six months as dependent factor

Characteristic	Odds ratio	95% CI
Presenting heart rhythm VF	0.4	0.1 to 1.2
Age	1.1	1.0 to 1.1
APACHE II score	1.1	1.0 to 1.2
SOFA score on admission	1.3	1.0 to 1.6
Spontaneous hypothermia	2.6	1.1 to 5.9
Spontaneous hypothermia adjusted for age, APACHE II score and SOFA score	3.8	1.3 to 11.0

CI, confidence interval; VF, ventricular fibrillation; APACHE, Acute Physiology And Chronic Health Evaluation; SOFA, Sequential Organ Failure Assessment.

## Discussion

In this observational cohort study among patients resuscitated for primary cardiac arrest, an unfavorable neurological outcome six months after ICU admission occurred significantly more frequently in patients with spontaneous hypothermia on ICU admission. Age, APACHE II score, SOFA score and spontaneous hypothermia on ICU admission all had an increased odds ratio for an unfavorable neurological outcome after six months. Among these four parameters, spontaneous hypothermia was the strongest independent predictor of unfavorable outcome. This was even more remarkable since predicted mortality calculated from the APACHE II score was significantly lower in patients with spontaneous hypothermia. In the subgroup of patients resuscitated outside the hospital, spontaneous hypothermia on ICU admission was an even stronger independent predictor of unfavorable neurological outcome than in the complete cohort.

To our knowledge, this is the first study analyzing the influence of ICU admission temperature on outcome. The result of our study is remarkable, as one might have expected a more effective cerebral protection in patients with spontaneous hypothermia, since the time needed to reach the target temperature during induced mild hypothermia was reduced in these patients. However, spontaneous hypothermia might represent a different clinical entity than induced mild hypothermia. Indeed, it is well-known that in patients with trauma and sepsis, spontaneous hypothermia on ICU admission is associated with a worse outcome [3,4]. In trauma patients, spontaneous hypothermia occurs in more severely injured patients and might therefore be caused by disrupted homeostasis and depletion of energy stores occurring during shock [5]. Indeed, at a cellular level, heat is produced by the hydrolysis of ATP to ADP and the anaerobic metabolism occurring in shock states may lead to a dramatic decrease in ATP production. Spontaneous hypothermia in combination with coagulopathy and acidosis is therefore considered as a lethal triad in trauma patients [5]. In septic patients presenting with hypothermia, mortality is approximately twice as high as in patients without hypothermia [6]. This increased mortality has been attributed to cerebral dysfunction in the setting of the multiple organ dysfunction syndrome [7].

It is conceivable that body temperature on admission can be used as a sign of the ability of the body to maintain temperature homeostasis. Under normal circumstances, body temperature is mainly regulated by the warm-sensitive neurons in the preoptic area of the hypothalamus [8]. When the temperature in the preoptic area decreases, inhibitory signals from the warm-sensitive neurons are blocked, leading to shivering. It is conceivable that in patients with spontaneous hypothermia after resuscitation, the mechanisms of cerebral thermoregulation are impaired due to ischemia and reperfusion injury, and that spontaneous hypothermia should therefore be interpreted as a sign of cerebral injury. To confirm this hypothesis, a prospective study should be performed.

A limitation of our study is that we did not record the interval between circulatory arrest and return of spontaneous circulation, the parameter repeatedly identified as a strong outcome predictor in patients after cardiac arrest [9,10]. As the current study was not performed prospectively, the time to return of spontaneous circulation was not systematically recorded in the patient files and was therefore difficult to retrieve in a reliable manner. Another limitation of our study is that we did not analyze other reasons for hypothermia, such as ambient temperature on the site of resuscitation or in the ambulance.

## Conclusions

This observational cohort study demonstrates that spontaneous hypothermia on ICU admission after resuscitation for primary cardiac arrest was associated with an unfavorable neurological outcome, possibly due to more extensive brain injury. Because of its limited specificity, spontaneous hypothermia cannot be used as an individual predictor. However, its presence after resuscitation may be used as an alert for an increased risk of unfavorable neurological outcome.

## Key messages

• Among patients resuscitated for primary cardiac arrest, an unfavorable neurological outcome after six months occurred more frequently in patients with spontaneous hypothermia on ICU admission (69% versus 50%, *P *= 0.05).

• Spontaneous hypothermia on ICU admission was the strongest predictor of unfavorable neurological outcome after six months (OR 2.6, 95% CI 1.1 to 5.9).

• After adjustment for age, APACHE II and SOFA scores, the influence of spontaneous hypothermia on neurological outcome was even stronger (OR 3.8, 95% CI 1.3 to 11.0).

## Abbreviations

APACHE: acute physiology and chronic health evaluation; CI: confidence interval; GOS: Glasgow Outcome Scale; ICU: intensive care unit; IQR: interquartile range; OR: odds ratio; PDMS: Patient Data Management System; SOFA: sequential organ failure assessment.

## Competing interests

The authors declare that they have no competing interests.

## Authors' contributions

AdH and LR retrieved the data and built the database. AdH performed the analysis and wrote the manuscript drafts. ACdP designed the study and coached the analysis. JB performed the statistical analysis and MJS and JH coached the analysis and supervised the project. All authors contributed in the writing and the critical appraisal of the manuscript. All authors read and approved the final manuscript.
